# Cryopreserved amniotic membrane and umbilical cord particulate for managing pain caused by facet joint syndrome: A case series

**DOI:** 10.1097/MD.0000000000014745

**Published:** 2019-03-08

**Authors:** Daniel S. Bennett

**Affiliations:** Denver Pain and Spine PC, Golden, CO.

**Keywords:** amniotic membrane, facet joint, facet joint syndrome, intra-articular injection, lower back pain, umbilical cord

## Abstract

Treatment of back pain due to facet joint syndrome has been a challenge for physicians since its recognition ∼80 years ago. Intra-articular injections of steroids, local anesthetics, and phenol have been widely adopted despite their known shortcomings. Recently, intra-articular injection of amniotic membrane-umbilical cord (AMUC) has been utilized in various orthopedic indications, including those involving synovial joints, due to its reported anti-inflammatory properties. Herein, use of AMUC for facet joint syndrome was evaluated.

A single-center case series was conducted on patients presenting with pain caused by facet joint syndrome, confirmed by single blocking anesthetic injection and treated using a single intra-articular injection of 50 mg particulate AMUC (CLARIX FLO) suspended in preservative-free saline. Patient reported back pain severity (numerical scale 0–10) and opioid use were compared between baseline and 6 months following treatment.

A total of 9 patients (7 males, 2 females), average age 52.1 ± 15.9 years, were included. Five patients with cervical pain had a history of trauma, 1 patient had suffered lumbar facet injury and 3 had degenerative lumbar facet osteoarthritis. All patients had severe pain prior to injection (8.2 ± 0.8) and 4 (44%) were taking opioids (>100 morphine milligram equivalents). Six-month post-treatment, average pain had decreased to 0.4 ± 0.7 (*P* <.05). All patients had ceased use of prescription pain medications, including opioids. No adverse events, repeat procedures, or complications were reported.

Intra-articular injection of AMUC appears to be promising for managing facet pain and mitigating opioid use. Further investigation with larger sample size is warranted.

## 1 Introduction

Chronic back pain is the main presenting complaint among patients seen by neurosurgeons and orthopedic surgeons with a prevalence ranging from 60% to 90%.^[1,2]^ Studies have shown ∼82% of these cases are attributed in part to the facet (zygapophysial) joints, which are the only true synovial joints of the spine.^[2]^ Just like joints of the knee, facet joints consist of a synovial capsule, synovial membrane, hyaline cartilage, and subchondral bone allowing for tension/compression resistance and facilitation of mobility.^[1,3]^ Stressed facet joints, degenerative arthritic changes, and muscle imbalances are all factors that contribute to a phenomenon known as facet joint syndrome.^[2,4]^

Facet joint syndrome often results from trauma, arthritis, chondromalacia, segmental instability, and degenerative changes which induce inflammatory mediators.^[2,5]^ Unfortunately, pain is highly sensitive and intensified in this area due to its dense innervation. Diagnosis is typically made clinically as well as through exclusion of other origins of back pain. The main symptom experienced with facet joint syndrome involves pain that increases with stress, exercise, extension of the spine, and rotational motions. Current conservative treatments for facet joint syndrome involve intra-articular injections of steroids, local anesthetics, and/or phenol, with steroids being the most popular agent used.^[1,2,6]^ Between 1994 and 2001, facet joint injections increased by 231% among Medicare patients.^[7,8]^ Widespread use of steroids continues despite variable clinical effectiveness and associated complications such as weight gain, hypertension, osteoporosis, insomnia, and psychosis.^[7–11]^ Thus, there remains unmet clinical need for a safer and more effective treatment for facet joint syndrome.

Amniotic membrane (AM) and umbilical cord (UC) tissues are placental tissues that can be processed using cryopreservation so as to retain key biological and structural components of the innate tissue.

(1) These tissues have been demonstrated to have both anti-inflammatory and anti-scarring properties in vitro and in vivo which has led to their use in many clinical orthopedic procedures.
(2) For example, intra-articular injection of particulate AMUC has been shown to improve pain in symptomatic knee osteoarthritis (OA), and to prevent cartilage destruction in a rodent model of induced OA.^[12,13]^

To assess the benefits of intra-articular AMUC in a pain management practice, a retrospective chart review was undertaken to evaluate symptomatic pain relief in patients suffering facet joint syndrome.

## 2 Methods

The objective of the case series was to evaluate the safety and effectiveness of injection of particulate AMUC for the treatment of facet pain. This case series was approved by the Institutional Review Board and conducted in accordance with the Helsinki Declaration as revised in 2000. Patient's charts were reviewed if they underwent intra-articular AMUC injections for facet syndrome by the author between January and October 2017 inclusive. The inclusion criteria were age 18 to 85 years, and at least 6 months of follow up data.

Diagnosis of facet joint syndrome was routinely performed as follows: flexion–extension radiographs were evaluated first to rule out dynamic instability or listhesis. Next, patterns of referred pain were noted, and range of motion was assessed through flexion, extension, lateral bending, and rotation. When the facet joint was identified as the primary pain source, intra-articular anesthetic injections were performed under fluoroscopy using either 0.5% lidocaine or 0.5% bupivacaine. Facet joints in the lumbar region were injected with a volume of 1.0 mL, while those in the cervical region were injected with a volume of 0.5 to 0.9 mL depending on the level. The patient was then asked to perform normal daily activities to trigger the pain and fill out a pain diary to determine when the pain occurred. This was then compared to the time period the local anesthetic is known to wear off. Concordance of the return of pain with the known duration of effect confirmed the diagnosis.^[11,14]^ Forty-eight hours following confirmation, each patient underwent injection in each joint (per level) of 50 mg AMUC (CLARIX FLO, TissueTech, Inc., Miami, FL) suspended in 0.5 mL of preservative-free saline. This dose was recently shown to attenuate pain in knee OA.^[13]^ A total of 0.4 mL was injected into the zygapophysial intra-articular space while 0.1 mL was injected around the inferior capsule area. Patients were allowed to return immediately to normal activities.

Medical records were reviewed for demographic variables (gender, age, body mass index (BMI)), diagnosis, opioid use, and patient subjective reported pain, which was obtained prior to and after diagnostic injections, 8 weeks after treatment and at 6 months post-treatment Pain is routinely evaluated using an 11-point numerical pain rating scale (0–10), where 10 represents the most severe pain and 0 represents no pain. Outcome measures were collected pre-injection and 6 months following intra-articular injection. The occurrence of complications, adverse events, or repeated interventions was also assessed for safety. Standard motor, sensory and reflex neurological evaluations were routinely performed.

### 2.1 Statistical analysis

All data were recorded using an Excel spreadsheet (Microsoft Corporation, Redmond, Washington). Descriptive statistics (median, range, mean ± standard deviation) are provided for patient demographics and pain scores. Change from baseline in pain score was evaluated using Wilcoxon Signed Rank Test. A *P* value of less than .05 was considered statistically significant.

## 3 Results

Among 30 patients treated during the designated timeframe, 9 (7 males and 2 females) met the criteria of age and length of follow-up (Tables 1 and 2). Cervical facet joints were affected in 5 patients, while lumbar facet joints were affected in 4 patients. The etiology of the facet joint was traumatic in 6 patients and OA in 3 patients. One patient aged 81 years-old had a 30-year history of lumbar OA prior to treatment with AMUC.

**Table 1:**
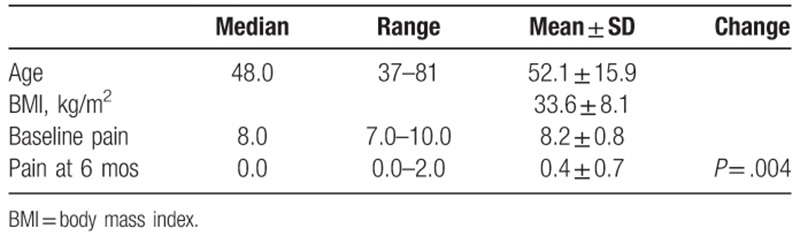
Baseline demographics and outcomes.

**Table 2:**
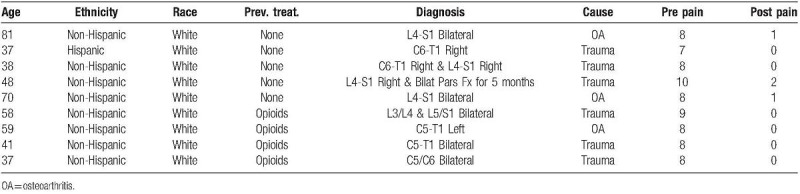
Individual Patient Data.

Before AMUC, all patients were clinically diagnosed with facet joint syndrome and had reported severe pain for an average duration of 25 ± 12 months excluding the aforementioned patient who had suffered lumbar pain for 30 years. All patients complained of pain despite oral administration of NSAIDS and acetaminophen. Four of the 9 patients (44%) were taking opioid medication (above 100 morphine milligram equivalents per day, 2 of which were oxycodone) at the time of the AMUC injection. After receiving AMUC injection, the patient's reported an average 94.6% decrease in pain (*P* = .004). Furthermore, no repeated interventions were required and all patients had ceased use of prescription pain medications. No adverse events or complications occurred over the 6-month follow up period.

## 4 Discussion

Treatment for chronic back pain resulting from facet joint syndrome is one of the main challenges faced by physicians today. There is no truly effective “gold standard” interventional therapy, leaving patients with significant disability, as well as extraordinary health care and societal costs.^[15]^ Intra-articular injection with steroids is a widely utilized approach intended to mitigate this issue, however, there is much controversy on its widespread use.^[1,2,6]^ For instance, in a double-blind, randomized, placebo-controlled trial, Carette et al^[11]^ found no short- or long-term improvement from intra-articular steroids. Other studies have found the same lack of benefit when compared with placebo injections.^[16]^ Systematic reviews of the literature find no compelling evidence of long term benefit, concluding that these injections are not clearly effective.^[9–11,17–19]^ In the present case series, intra-articular facet injection of AMUC led to significant pain relief for at least 6 months. The overall decrease in pain was 94.6%, which is comparatively better than what has been reported with corticosteroid use or radiofrequency nerve ablation.^[9–11,17–19]^ Recent studies have shown radiofrequency ablation to provide a ∼70% relief for more than 6 months with a mean duration for relief of 10.5 months.^[20]^ It will be of interest to examine pain reduction at 1 year following the AMUC therapy.

The therapeutic effect of AMUC in facet joint syndrome may be due to the tissue's anti-inflammatory properties. In previous studies, inflammation has been shown to be associated with facet joint syndrome as inflammatory cytokines in the facet joint is found at high levels that may contribute to pain.^[2,5]^ Hence, AMUC's anti-inflammatory actions may attenuate the inflammatory mediators and relieve pain. More specifically, AMUC tissues have been shown to downregulate expression of pro-inflammatory cytokines such as TNF-α and IL-6, induce apoptosis of pro-inflammatory cells such as activated neutrophils and M1 macrophages and promote polarization of anti-inflammatory cells such as M2 macrophage.^[21]^ Although AMUC contains a myriad of growth factors, cytokines, and extracellular matrix components, the aforementioned therapeutic effects have primarily been shown to come from a molecular complex known as HC-HA/PTX3.^[21]^ Pre-clinical and clinical studies have confirmed these therapeutic benefits in diseased joints where particulate AMUC attenuated progressive cartilage degeneration and provided pain relief of symptomatic knee OA.^[12,13]^ Considering that the knee and facet share similar anatomical features and osteoarthritic changes have been a universal finding in facet degeneration,^[4]^ it is not surprising the current case series show similar therapeutic effects as previously seen in those studies.

A novel finding was that patients relying on opioids for pain relief were able to entirely discontinue their use after AMUC injection. While opioids can provide short-term relief, they are also known to have a high abuse potential and produce several undesirable effects such as nausea, vomiting, constipation, somnolence, and respiratory depression. Each day, about 115 Americans are said to die from unintended opioid overdose, with 40% of those deaths originating due to prescribed opioid use.^[22,23]^ Consequently, in October 2017, the opioid crisis was declared as a national public health emergency.^[24]^ As such, there remains an unmet clinical need for a novel, non-opioid alternative which provides a more effective treatment to prevent opioid abuse, dependence, and addiction in pain management. Further controlled studies evaluating different dosages are necessary to determine if AMUC injection might be a safer non-opioid alternative to treat pain derived from the facet joint syndrome.

## Author contributions

**Conceptualization:** Daniel S. Bennett.

**Data curation:** Daniel S. Bennett.

**Formal analysis:** Daniel S. Bennett.

**Investigation:** Daniel S. Bennett.

**Methodology:** Daniel S. Bennett.

**Project administration:** Daniel S. Bennett.

**Resources:** Daniel S. Bennett.

**Supervision:** Daniel S. Bennett.

**Validation:** Daniel S. Bennett.

**Writing – original draft:** Daniel S. Bennett.

**Writing – review & editing:** Daniel S. Bennett.
